# Luminal Fluid Motion Inside an In Vitro Dissolution Model of the Human Ascending Colon Assessed Using Magnetic Resonance Imaging

**DOI:** 10.3390/pharmaceutics13101545

**Published:** 2021-09-23

**Authors:** Connor O’Farrell, Caroline L. Hoad, Konstantinos Stamatopoulos, Luca Marciani, Sarah Sulaiman, Mark J. H. Simmons, Hannah K. Batchelor

**Affiliations:** 1 School of Chemical Engineering, University of Birmingham, Birmingham B15 2TT, UK; Konstantinos.x.Stamatopoulos@gsk.com (K.S.); M.J.Simmons@bham.ac.uk (M.J.H.S.); 2 Sir Peter Mansfield Imaging Centre, School of Physics and Astronomy, University of Nottingham, Nottingham NG7 2RD, UK; Caroline.L.Hoad@nottingham.ac.uk; 3 National Institute for Health Research (NIHR), Nottingham Digestive Diseases Biomedical Research Centre, Nottingham University Hospitals NHS Trust, University of Nottingham, Nottingham, UK; Luca.Marciani@nottingham.ac.uk (L.M.); Sarah.Sulaiman@nottingham.ac.uk (S.S.); 4 Biopharmaceutics, Pharmaceutical Development, PDS, MST, RD Platform Technology & Science, GSK, David Jack Centre, Ware SG12 0DP, Hertfordshire, UK; 5 Strathclyde Institute of Pharmacy and Biomedical Sciences, University of Strathclyde, Glasgow G4 0RE, UK; Hannah.Batchelor@strath.ac.uk

**Keywords:** dynamic colon model (DCM), large intestine, colon, colon-specific drug formulations, colonic flow, phase contrast cine-MRI, MR tagging, colonic mixing

## Abstract

Knowledge of luminal flow inside the human colon remains elusive, despite its importance for the design of new colon-targeted drug delivery systems and physiologically relevant in silico models of dissolution mechanics within the colon. This study uses magnetic resonance imaging (MRI) techniques to visualise, measure and differentiate between different motility patterns within an anatomically representative in vitro dissolution model of the human ascending colon: the dynamic colon model (DCM). The segmented architecture and peristalsis-like contractile activity of the DCM generated flow profiles that were distinct from compendial dissolution apparatuses. MRI enabled different motility patterns to be classified by the degree of mixing-related motion using a new tagging method. Different media viscosities could also be differentiated, which is important for an understanding of colonic pathophysiology, the conditions that a colon-targeted dosage form may be subjected to and the effectiveness of treatments. The tagged MRI data showed that the DCM effectively mimicked wall motion, luminal flow patterns and the velocities of the contents of the human ascending colon. Accurate reproduction of in vivo hydrodynamics is an essential capability for a biorelevant mechanical model of the colon to make it suitable for in vitro data generation for in vitro in vivo evaluation (IVIVE) or in vitro in vivo correlation (IVIVC). This work illustrates how the DCM provides new insight into how motion of the colonic walls may control luminal hydrodynamics, driving erosion of a dosage form and subsequent drug release, compared to traditional pharmacopeial methods.

## 1. Introduction

Knowledge of luminal flow inside the human colon remains elusive, as this anatomical region is difficult to access and current techniques are generally invasive. Improved knowledge would enable a greater understanding of colonic physiology and pathology, enhancing the capability to design new or improved colon-targeted drug delivery systems. Targeted drug delivery to the ascending colon (AC) offers a promising opportunity for local administration of effective therapeutics for a range of conditions, such as inflammatory bowel disease, colon cancer and irritable bowel syndrome, all of which have enormous and increasing prevalence across the globe [1,2,3]. In the case of colorectal cancer (CRC), it is the fourth most commonly diagnosed and third most deadly cancer worldwide [1]. The combination of enhanced biorelevant in vitro and in silico dissolution testing could streamline the research and development process for new colon-targeted formulations [4,5]. In silico models that can simulate luminal flow inside the colon enable the investigation of colonic drug delivery systems to interpret how flow parameters can affect the breakdown of different formulations and API release [6,7]. Formulations can then be optimised by the use of quantitative simulations of how luminal flow affects mass transport phenomena of the API, including release and dissolution rates and distribution via convective transport inside the lumen.

Manometry techniques are used to measure intraluminal pressure activity during contractions of the colonic wall, with significant advances being made using high resolution manometry in recent years [8,9,10,11]. However, little information is available pertaining to how the colonic contents move and particularly, changes in movement based on motility patterns associated with both normal and abnormal colon pathophysiology [12]. For example, unsynchronised or reduced motility patterns of the colon wall (as a result of a functional gastrointestinal disorder, FGID), may not be effective for mixing and propulsion of the colonic contents. Colonic mixing phenomena occur in the AC (before significant dewatering occurs), which is particularly difficult to access using manometry, and highly invasive. In this region, it is possible that the hydrodynamics can be affected by disorders that cause extreme variances in physical properties of the chyme. The influence of disease affecting the AC can therefore have a significant impact on the delivery of colon-targeted dosage forms. Reduced contractile activity of the AC walls may lessen the impact of normal and shear forces arising from fluid motion and direct contact with the colonic walls. Therefore, a technique to measure motion of the colonic walls and contents in conditions that incorporate extremes of disease would have great value in the development of patient-centric colonic delivery systems.

Magnetic resonance (MR) tagging, a noninvasive and nonionising imaging technique which is commonly used to assess cardiac function, has recently been applied to assess gastrointestinal motility [13,14,15,16]. MR tagging involves deliberately altering the MR imaging (MRI) signal along parallel lines or ‘tags’ in an organ inside the body, at a given time before acquiring an image. A comb of parallel black tags is ‘drawn’ on the tissue and if the tissue moves between the tagging process and the action of taking the MRI image, the black lines are observed to move with the tissue. This is captured on the MRI image as deformation of the black lines in the direction of motion, with the amount of deformation being proportional to the degree of motion. MR tagging velocimetry then involves the tracking of a tagged material or volume of fluid to estimate the local velocity from the displacement of tags between frames. It has been used to measure the velocity in fluid flows, primarily in blood vessels [17] and more recently in engineering systems [18,19]. Pritchard et al. [20] and Wilkinson-Smith et al. [16] applied an MR tagging technique to the flow of the contents of the human AC. A principal output of their work was the definition of a coefficient of variance (CoV) parameter, that may successfully discriminate between healthy and constipated subjects based on the degree of mixing-related motion that occurred.

**Figure 1:**
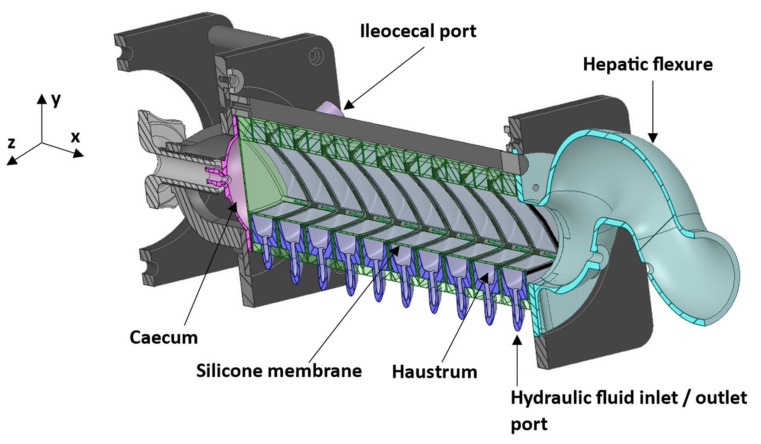
Schematic cross-sectional view of the Dynamic Colon Model (DCM). The DCM has a segmented appearance reflecting that of the human ascending colon: segment 1 is adjacent to the *caecum*, through to segment 10 adjacent to the *hepatic flexure*.

This research aims to use MR tagging to visualise, assess and discriminate between different motility patterns applied to an in vitro model of the human AC, the dynamic colon model (DCM). The DCM (Figure 1) replicates the anatomy, physical pressures and motility patterns of the human AC [21,22,23]. Precise replication of well controlled, repeatable motility patterns and therefore hydrodynamic conditions permits an intensive analysis which is not possible in vivo as colonic motility is inherently erratic. Therefore, good performance in vitro would provide further validation for the CoV parameter in addition to enabling a rapid estimation of the intensity of hydrodynamic activity that a dosage form may be subjected to inside the luminal environment during dissolution testing. The biorelevant DCM is a valuable dissolution testing apparatus, since existing pharmacopeial dissolution testing apparatuses do not mimic the hydrodynamics of the AC [22,24], as they were designed with simple architecture and mixing mechanisms to satisfy the need for the batch-to-catch quality control of dosage forms [25].

The DCM also enables tight control of biorelevant parameters, such as the viscosity and volume of the lumen contents, that may be affected by functional gastrointestinal disorders (FGIDs) [26] and are pivotal in the release of the API from a dosage form [27,28,29]. Different motility patterns can be applied reproducibly to investigate a range as well as extremes of motion; thus, the sensitivity of a formulation to these extremes can be understood which can help to understand the variability in product performance that may exist in health and disease. Thus, the biorelevance of the system can encompass the variability that may be observed in vivo by accurate replication of a range of motility patterns. The interplay between colonic environmental parameters and the applied motility pattern on the motion of the luminal contents can be evaluated using MR tagging within the DCM. Again, this is not possible within the in vivo colonic environment, which is also subject to the inherent variability of in vivo measurements and effects of respiratory motion. Therefore, validation of an MRI technique using the DCM provides researchers and clinicians an additional tool to understand how underlying abnormalities affect fluid flow within the colon.

This study also extends analysis (of the same tagged dataset) using MR tagging velocimetry, to explore the in-plane measurement of velocity of the contents inside the DCM lumen arising from motility patterns that mimic antegrade cyclic propagating pressure waves (CPPWs). CPPWs are among the most abundant forms of motility observed in the unstimulated human AC [30]. Furthermore, the same approach will be applied to measure velocities of the contents of the human AC using a tagged sequence previously published by Pritchard et al. [20]. This will facilitate a direct comparison of luminal flow in the colonic environment with its in vitro counterpart. Additional understanding of the similarities and differences between in vitro and in vivo systems could be valuable to inform in vitro in vivo evaluation (IVIVE) or in vitro in vivo correlation (IVIVC) studies using the DCM. These can reduce the number of in vivo studies, that are costly in terms of both time and money, during the development of new pharmaceutical formulations. However, similarly to pharmacopeial dissolution apparatuses, the DCM is limited in that it cannot currently model absorption kinetics. Therefore, assumptions need to be made regarding permeability and dissolution–absorption relationships according to data from cellular models, such as Caco-2 or colonoids.

Phase contrast (PC) cine-MRI, described in detail in [31], is also used to measure blood flow [32]. To date, no nuclear magnetic resonance (NMR) velocimetry studies have been carried out on the human AC, nor on phantoms that closely replicate the geometry and flows inside the colonic environment. Both MRI techniques enable visualisation of the motion of the DCM wall and lumen contents. This work implements both techniques to measure the in-plane (MR tagging) and through-plane (PC cine-MRI) velocity of the contents of the DCM lumen synchronously with the movement of the DCM colonic walls; to develop an understanding of the hydrodynamic conditions a dosage form is subjected to during dissolution testing inside the DCM.

## 2. Materials and Methods

### 2.1. Dynamic Colon Model and Lumen Media

The DCM was filled with liquid media and placed in the MRI scanner in the supine patient position. The liquid media were chosen to mimic a biorelevant viscosity based on previous studies [21,22,27], by using aqueous solutions of NaCMC (700,000 Mn); denoted as LOVIS (low viscosity, 0.25% (w/v) NaCMC, 13 mPa s) or HIVIS (high viscosity, 0.50% (w/v) NaCMC, 98 mPa s). Media volumes varied from 150 mL to 200 mL. The wall motility waves applied in this study were antegrade contractile waves travelling the entire length of the DCM (20 cm), in line with the default pattern previously applied [21,22]. Propagation speeds of 0.8 cm·s^−1^ and 0.4 cm·s^−1^ were used. The former is the average propagation velocity of a cyclic antegrade wave in the fed state in the colon and the latter is the average propagation speed of a high amplitude propagating contraction (HAPC) [30]. The occlusion degree was fixed at 60 ± 5% for each pattern, higher than in previous reports, to further explore the graduation of fluid flow with wall contraction [22].

### 2.2. MR Protocol

Scanning was carried out using a 3T Philips Ingenia Widebore scanner (Philips, Best, The Netherlands). Localiser scans were carried out prior to the tagging and PC scans for placement of these sequences across the DCM.

Firstly, motion of the contents of the DCM lumen was visualised using a tagged balanced turbo field echo (bTFE) sequence. This sequence saturated predetermined regions of fluid inside the DCM by applying a radiofrequency pulse to null the signal of these regions. This resulted in dark horizontal stripes (tags) being superimposed onto the images, with 12 mm spacing between the centres of consecutive tags. The delay of 250 ms between the application of the tag lines and acquisition of the image allowed movement within the DCM lumen to be detected. The tags were approximately 5 mm thick. Sharpness and width of the tag lines are influenced by the time it takes to run the tagging pulses and this sequence uses a slightly longer pulse train than the default set for cardiac applications, which improves the definition of the edges of the tag. A shim box was placed over the area of interest to reduce the susceptibility to artefacts in the region in vitro, in line with the in vivo scan [20]. This sequence had a repetition time (TR)/echo time (TE) of 2.44/1.22 ms, a flip angle (FA) of 45°, with a single sagittal slice, thickness of 15 mm, a field of view (FOV) of 259 mm (anterior–posterior (AP)), and 330 mm (head-feet (HF)) with a reconstructed resolution of 0.98 × 0.98 mm^2^. In total, 100 scans were acquired at 600 ms intervals. Important imaging parameters are summarised in Table 1, including those used for the in vivo study, which the DCM sequence was based upon. After acquisition of a tagging sequence, a rest period of 10 s was allowed for the lumen media to stabilise before running subsequent sequences.

Secondly, PC cine-MRI scans were acquired using a sequence modified from a standard PC flow sequence that normally acquires multiple flow measurements in arteries (and veins) across the cardiac cycle. This method is described in detail in [18]. For this study, a single fast field echo (FFE) image was generated using flow-sensitive gradients with a TE = 7.6 ms and TR = 9.2 ms. Each image of 101 × 101 voxels was generated with an in-plane reconstructed resolution of 1.136 × 1.136 mm and a slice thickness of 8 mm. This scan was then repeated over a 60 s period with a temporal resolution of 2 s resulting in a set of 30 images for each parameter combination. Three different slice locations along the length of the DCM were used sequentially, with 10 s rest periods between scans to investigate the spatial variation of the flow induced. The locations were at segments 2, close to the *caecum*, 6, the midpoint and 10, the *hepatic flexure* (see Figure 1). Following completion of all spatial locations for the default motility pattern, the protocol was repeated for the slower CPPW. After completion of all scans, media volume and/or media type (LOVIS or HIVIS) were changed, and the protocol repeated. Flow was encoded only in the streamwise direction (*x*-axis). To avoid aliasing, maximum velocities were encoded at ±3 cm·s^−1^, 50% higher than the fastest propagating wave speed of 2 cm·s^−1^ which has been shown by Stamatopoulos et al. [22] to be close to the maximum media velocity inside the lumen. Positive and negative velocities represent flow along the *x*-axis towards the *hepatic flexure* and *caecum* (depicted in Figure 1), respectively.

### 2.3. MR Data Analysis

**Figure 2:**
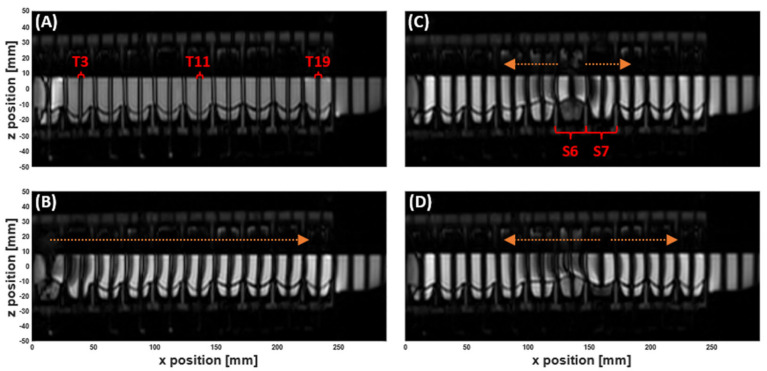
MR Tagging applied to the dynamic colon model (DCM) filled with 200 mL LOVIS fluid. ‘Tags’ are the dark stripes across the images. The DCM is orientated such that the *caecum* is at *x* = 0 mm, and the *hepatic flexure* begins at *x* = 250 mm. (**A**) Coronal image showing no movement inside the DCM before motility is initiated. The tags are straight, parallel and intact. (**B**) Tagged image during contraction of the first DCM segment. Tags are shifted in the positive *x*-direction showing a wave of antegrade flow travelling through the lumen. (**C**) and (**D**) Tagged images of the DCM during contraction of segment 6, showing antegrade flow in the immediately downstream tag at early stages of contraction (**C**) and stronger retrograde flow in all adjacent tags (**D**). The arrows drawn onto the images are to aid visualisation of the direction of tag displacement and therefore motion of the contents. Tags (T) 3, 11 and 19, and segments (S) 6 and 7 are labelled as these were the focus of velocimetry studies in Section 3.2 and Section 3.3.

Any movement of the lumen contents during the delay between the application of a tag and image acquisition changes the position of the tag lines on the image. However, if no motion occurred during the complete scan, the full set of sequential tagged images would be identical, with all tag lines remaining straight (as seen in Figure 2A). Motion of the luminal contents, in any direction, leads to changes in the signal intensity in the tagged contents from frame to frame. This variation forms the basis of the method proposed to assess motion inside the DCM.

**Figure 3:**
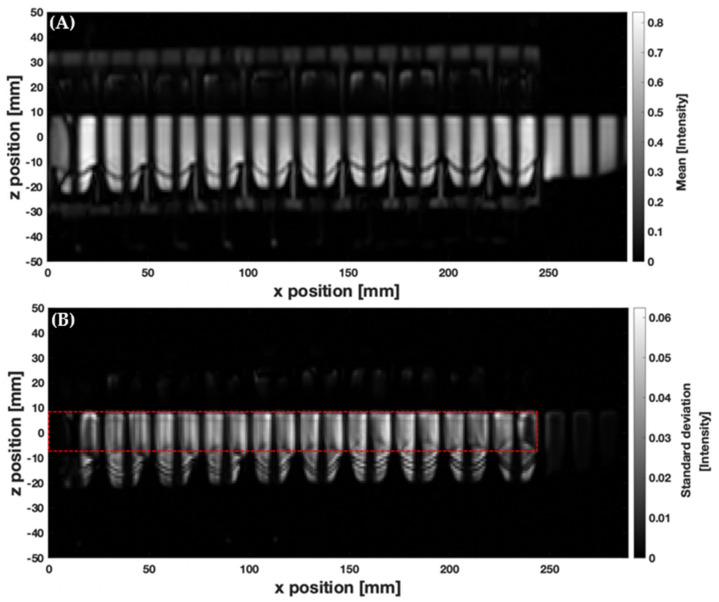
Processed images from tagged cine-MRI data showing (**A**) a mean voxel intensity map and (**B**) the corresponding voxel standard deviation map (calculated over 100 dynamic images). The red dashed line depicts the ROI, R, that encloses the DCM lumen.

To measure motion of the contents of the human AC, Pritchard et al. [20] analysed standard deviation maps within a region encompassing the AC, calculated over a sequence of tagged images. Applying this technique to the DCM, a region of interest (ROI), R, was drawn, enclosing the lumen at the neutral wall position (outlined in Figure 3B) to focus measurements on the motion of fluid inside the lumen rather than fluids inside the haustra, used for the pneumatic control of wall motion. Those voxels within R which experience variation in the signal intensity during the 60 s of data acquisition have a relatively large standard deviation of intensity, whereas static structures (DCM core structure) or motionless lumen contents have a standard deviation close to zero. Therefore, the resulting standard deviation map highlights any motion of the DCM lumen contents. The standard deviation map can also reveal where the motion is concentrated. The mean signal intensity (MIR) and standard deviation (STDEVR) maps were calculated using MATLAB (R2019B, The MathWorks, Inc., Natick, MA, US) over 100 frames. The coefficient of variation (CoV) for the tagged scan was then estimated from:

CoV = 100 × STDEV_R_/MI_R_ (1)

The predominant direction of flow (antegrade or retrograde) is revealed from the direction of tag displacement. If motility induces laminar flow inside the DCM or AC lumen, each frame would show a uniform displacement of each tag line, where tag displacement would be proportional to flow velocity. Velocity estimates (u) of lumen contents were obtained from tagged MRI images of the DCM acquired in this study, and from in vivo sequences previously published in the supplementary material of the Pritchard et al. [20] study. This was described by the measured displacement (Δx) of a point of the tag over a fixed period of time (Δt)—the delay of 250 ms between the application of the tag lines and acquisition of each image—as shown by Equation (2). Different parts of a tag can travel different velocities [33]. The average velocity of the tagged contents was calculated by measuring the displacement of the centroid of each tag between consecutive images, whilst the peak velocity was measured using the maximum displacement of each tag between consecutive images, i.e., tracking the displacement of the leading edge of each tag. Displacement could be accurately measured to within ± ½ voxel diameter, ±0.49 mm, which translates to an uncertainty of ±0.20 cm·s^−1^ associated with TOF tagging velocity measurements.

u_x_ = Δx/Δt (2)

The in vivo data available for assessment included one sequence with extreme motility obtained after stimulation by ingestion of a 500 mL dose of polyethylene glycol (Macrogol 3550) electrolyte solution (MOVIPREP^®^, Norgine Pharmaceuticals Ltd., Harefield, UK). Details of the participants are outlined in the study described in [20].

Using PC cine-MRI, the mean velocity of the DCM lumen contents was measured by taking the mean of all the weighted-average velocities measured in voxels that constitute the through-plane lumen cross-sectional flow area. Additionally, a second measure of the mean velocity was made by taking the mean of all the weighted-average velocities measured in voxels encompassed by the ‘central flow region’ of the lumen. This was to assess the impact of any stagnant regions of fluid close to the walls on the through-plane mean velocity. Furthermore, peak velocities were also measured by taking the mean of the 6 highest value voxels within each ROI. Due to the potential for high noise in individual voxel velocity measurements, PC cine-MRI peak velocity estimates should be made using several voxels, rather than just one [34]. The standard deviation of the mean velocity calculated using each ROI was considered to be the error associated with the respective PC cine-MRI mean velocity measurement.

### 2.4. Statistics

A three-way ANOVA was performed to investigate the main effects of volume, viscosity and wave speed, in addition to their interaction effects, on CoV. Data were tested for normality using the Shapiro–Wilk test, and homogeneity of variances using Levene’s test for equal variances. Post-hoc analysis employed Tukey’s honest significant difference (HSD) test to assess significant differences in the mean of CoV between groups (*p* < 0.05).

## 3. Results and Discussion

### 3.1. MR Tagging of Fluid Motion

The series of tagged images enabled visualisation and quantification of the motion of the DCM luminal contents, whilst simultaneously tracking the dynamic morphology of the interior DCM walls during motility, as shown in Figure 2 and Supplementary Materials Video S1. Prior to initiation of a motility pattern, the DCM was at its ‘baseline’ state, as shown in Figure 2A. The DCM walls were clearly distinguishable against the media in the lumen and fluid inside the haustra, without the need for additional contrasting agents. Tags were straight, parallel and intact, showing no motion. Contraction of the first segment close to the caecum, synchronous with the relaxation of the second segment, drove a wave of antegrade flow along the positive *x*-direction (aboral), as shown by displacement of the tags in Figure 2, also indicated by the direction of the arrow above (B). The initial wave front travelled the length of the DCM (25 cm), identified as all tags were displaced. This sequence of segmental motion demonstrates how the DCM reproduces the widely accepted law of the intestine, wherein synchronised constriction and relaxation of the lumen results in a peristaltic wave [22].

Wall motility generated both antegrade and retrograde motion of the contents, as expected from in vivo observations [20]. As a wall segment contracted, for example, S6 in Figure 2C, the tagged contents on either side were displaced in opposite *x*-directions. Upon reaching maximum contraction, the principal flow direction of all immediately adjacent tags switched to retrograde as shown in Figure 2D. Arrows have been drawn on the images above the displaced tags to aid the visualisation. The data from this full tagging sequence is shown in a Supplementary Materials Video S1.

Figure 3 shows the mean (A) and standard deviation (B) maps of voxel intensity over the duration of the slower CPPW inside the DCM, with 200 mL LOVIS fluid inside the lumen. These relate to movement and blurring of the tags and therefore mixing-related motion of the contents. For all media, the standard deviation of voxel intensity varied along the *x*-axis; therefore, the level of motion experienced by the contents is nonuniform along the *x*-axis. This complements findings from dissolution studies inside the DCM, where different drug concentrations are found at different sampling points along the *x*-axis [21]. Low levels of motion were observed close to the caecum (*x* = 0 mm), increasing along the *x*-axis of the DCM lumen to a peak just after the midpoint, before decreasing close to the hepatic flexure (*x* = 250 mm). This indicates that the efficiency with which the DCM mixes and transports the contents may be low close to the start and end of a CPPW, suggesting that a solid dosage form may experience a slower eroding activity and therefore release rate in these regions. In this study, the beginning and end of the CPPW were fixed at the mimic caecum and hepatic flexure, respectively. This was based on clinical findings that showed an antegrade contractile wave ending at the hepatic flexure [22]; however, in vivo contractions can start and end in different locations [30]. A limitation of the model is that the hepatic flexure in the DCM is a rigid body, whereas in vivo it contracts to aid transfer of contents from the AC to the transverse colon, which may account for less intensive mixing phenomena observed in the DCM. Moreover, in vivo the AC is regularly receiving chyme from the ileum through the ileocecal valve which may increase mixing activity, compared to the DCM which acts as a closed system, rather than a flow-through style apparatus.

The calculated average coefficient of variation (% CoV) for all tagged scans are shown in Table 2, along with the post-hoc analysis of main effects. Data were found to be approximately normally distributed using the Shapiro-Wilk test for normality, with homogeneous variances. Effects of media volumes on the degree of mixing were not statistically significant (*p* > 0.05). However, the motility pattern and media viscosity were found to have statistically significant effects on the degree of mixing (*p* < 0.05). Interaction effects were not tabulated as all were found to be insignificant (*p* > 0.05), indicating that there was no combined effect for the motility pattern, media viscosity and volume on the degree of mixing under the experimental conditions in this study. However, differences are expected to be more extreme over a broader parametric range.

The above findings demonstrate that the tagging technique can discriminate between motility patterns based on the degree of mixing-related motion (*p* < 0.05) inside the DCM and reinforce findings made in vivo [20]. The degree of mixing-related motion significantly increased with the propagation speed of the contractile wave, supporting results by Thorpe et al. [35], where the distribution of 5-aminosalicylic acid (5-ASA) concentration increased with elevated levels of simulated motility within a colonic model. Levels of colonic motility differ based on disease and prandial states in vivo. For example, patients with chronic idiopathic constipation exhibit reduced colonic motor activity [36,37], and the level of retrograde CPPWs increases in the fed state compared to fasted [30]. Thus, the DCM could be used to study relationships between the motion of the colonic walls and contents of the colon, and the resulting impact on drug release and distribution when replicating distinctive types of motility patterns from clinical observations. This information is essential to inform the disease-state-specific design of locally acting dosage forms for the colon, in addition to the administration recommendations for such a formulation (with or without a meal).

Tagging was also able to discriminate between media viscosities based on the degree of mixing-related motion (*p* < 0.05). This additional sensitivity enables further understanding of the pathophysiology of colonic flow and the mode of action of treatments for functional colonic diseases, such as laxatives. In silico modelling of tablet dissolution in the proximal colon has shown that the ability of a motility pattern to distribute a dissolved API along the proximal colonic axis is highly dependent on the viscosity of the contents [6]. Viscosity has also been widely shown to influence dissolution in vitro [21,27,38]. Therefore, tagging scans that can distinguish between the viscosity of the contents based on mixing-related motion may be useful to inform the effectiveness of how a locally acting drug may be distributed in the region containing the target area. This area is currently very poorly understood, and therefore additional information provided by tagging is valuable and could in turn aid the design and optimisation of patient-centred therapeutic formulations.

### 3.2. Velocimetry of Fluid Motion In Vitro Using MR Tagging

**Figure 4:**
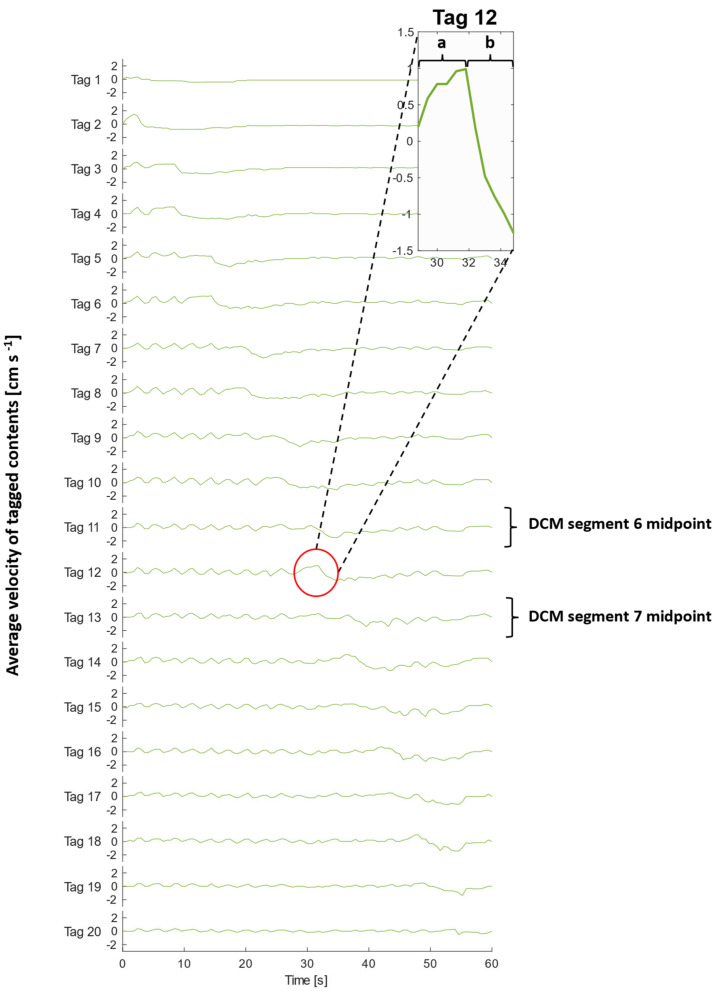
Average velocities of tagged DCM contents (200 mL LOVIS fluid) at each time point during a CPPW. Tag 1 is closest to the *caecum*, whilst tag 20 is closest to the hepatic flexure. The inset graph displays a magnified plot of tag 12 during (a) contraction of segment 6 and relaxation of segment 7 and (b) contraction of segment 7.

**Figure 5:**
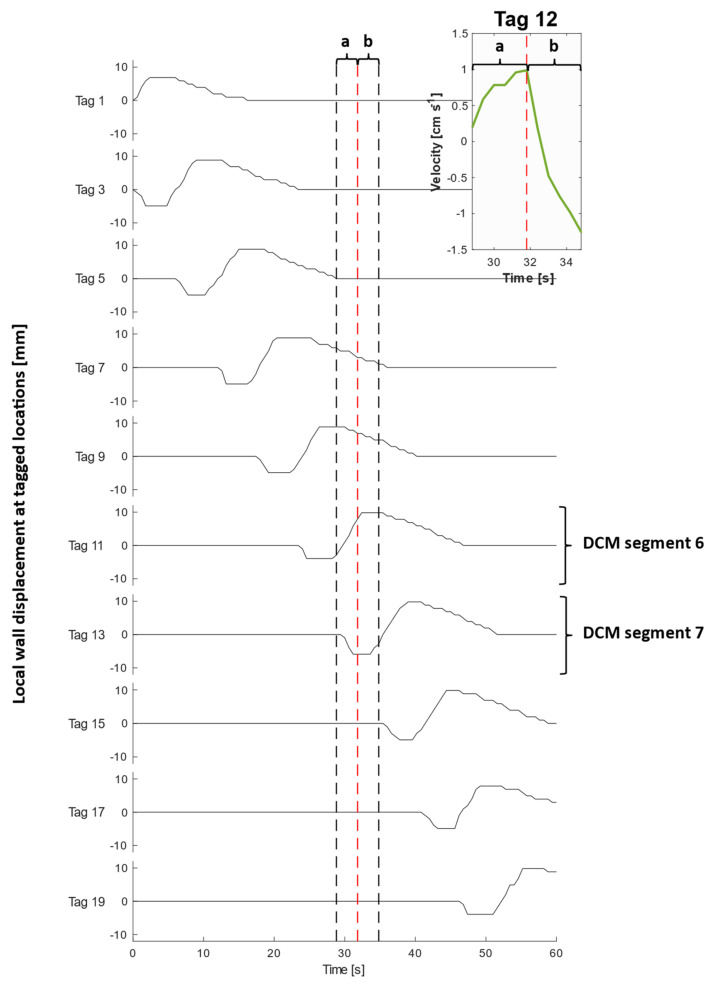
Local wall displacement at each tagged location during a CPPW. Note there is no local wall motion at the location of even-numbered tags due to rigid walls, which have hence been excluded from this plot. The inset graph displays a magnified plot of the velocity of the contents at tag 12 taken from Figure 4, during (a) contraction of segment 6 and relaxation of segment 7 and (b) contraction of segment 7. The black dashed lines bound the timeframe of the inset figure to aid visualisation of local wall motility surrounding tag 12 during the recorded flow event.

Figure 4 shows the measured average velocity of each MR tag over 100 consecutive images collected over the duration of the slow CPPW with 200 mL LOVIS fluid A inside the lumen. Figure 5 illustrates the local wall displacement at the location of odd-numbered tags, which were placed at the midpoint of each DCM segment, facilitating visualisation of how the contractile wave propagated along the *x*-axis of the DCM tube. For the slower CPPW, this analysis produced a quantitative overview of flow events along the entire DCM tube that occurred due to contractions in the walls of the DCM, enabling a comparison of flows induced by wall motility at different spatiotemporal locations along the in vitro colon model. There was no local wall motion at the location of even numbered tags as these were placed between segments where the walls are rigid, hence they were excluded from this plot.

Prior to motility of a given segment, the walls remained at the neutral position (0 mm displacement), whilst contents of the DCM lumen experienced a phasic cycling between low amplitude antegrade and retrograde velocities between the approximate limits of 0.80 and −0.40 cm·s^−1^ in a ‘to and fro’ motion. This showed that the media flow field was out of phase with the CPPW, i.e., a sloshing motion was observed, as reported in previous studies by Stamatopoulos et al. [21,22]. During motility, each segment followed an identical motion sequentially from segments 1–10, which consisted of a relaxation stage at 0.80 cm·s^−1^ to −3.20 ± 0.30 mm while the previous segment contracted, followed by a contraction stage where the haustra were inflated causing the walls to move at 0.80 cm·s^−1^ via the neutral position to reach a maximum displacement of 10.40 ± 0.60 mm. The wall remained at the maximum displacement for 2 s before relaxing to the neutral position at 0.18 cm·s^−1^.

An extended period of antegrade motion of the contents was observed for each tag that aligned with contraction of the immediately upstream segment and relaxation of the immediately downstream segment which began simultaneously, as is clear from Figure 5. This was followed by a sharp burst of retrograde flow during contraction of the immediately downstream segment. This pattern was expressed most clearly by even-numbered tags and is consistent with clinical data that showed fast retrograde flows during the relaxation of the ascending colon wall after propagation of an antegrade wave (video S2, taken from [20]). This implies that the position of the tagged luminal contents along the *x*-axis with respect to wall activity, shown in Figure 5, was decisive in determining the principal direction of motion. An example of this flow behaviour is illustrated in the inset plot of Figure 4 and Figure 5, showing the velocity profile of tag 12, which lies between segments 6 and 7 (see Figure 2 parts (A) and (B)), during contractile activity of those adjacent segments which are delineated by the dashed lines in Figure 5. Part (a) encloses the antegrade velocity increase that coincides with the wall contraction of segment 6, and wall relaxation of segment 7, whilst part (b) highlights the subsequent negative velocity jet as the wall contraction of segment 7 commenced. Even-numbered tags either side of a segment, where the walls were rigid and maintained a constant lumen diameter, consistently experienced higher magnitudes of velocity and more dramatically changing velocity profiles, compared with a seemingly dampened version of this profile for odd-numbered tags located in the centre of a segment where contractile activity occurs.

The highest average antegrade velocity was recorded to be 1.50 ± 0.20 cm·s^−1^ for tag 2 after contraction of the first segment. The average retrograde velocity peaked at −1.50 ± 0.20 cm·s^−1^ for tag 11 at time *t* = 34.8 s. Given the extent of retrograde activity, it would be expected that the net aboral propulsion of suspended fluid contents would be poor when subjected to the CPPW, similar to previous observations from studies that applied faster propagating waves in low viscosity media [21,22]. However, it is highlighted in Figure 4 and Supplementary Materials Video S1, that this motility pattern does not generate one continuous wave front in the contents that progress further along the *x*-axis of the DCM with each contraction, as in recent in silico studies [39]. This was expected as the CPPW was not a fully occluding event, therefore backflow was more prevalent post-contraction.

Identification of general flow and mixing patterns inside the lumen are valuable for the design of colon-targeted drug delivery systems. Many dosage forms that target the colon control drug release via erosion of the coating materials, which is controlled by luminal flow. The shear rates at the surface of these solid materials control the rate of erosion and hence drug release; therefore, understanding flow patterns is of high importance when designing medicines. For example, a recent simulation of drug release from a solid dosage form in the human ascending colon showed that motility patterns which provoked frequent single peaks in shear stress exerted on the surface of the dosage form, appeared to accelerate the release of the API [6]. Thus, any advances in knowledge of flows inside the DCM during the replication of motility patterns observed in healthy and diseased populations, enhances the value of this tool in the development of patient-centric formulations.

However, analysis proved challenging for the faster CPPW, as tags overlapped with one another, making it difficult to clearly distinguish the displacement of one tag from another without simplification that would greatly interfere with the data. Therefore, the tagging velocimetry methodology equipped with this particular parameter setup in this investigation was only suitable for analysis of the slower motility pattern. In the future, this could be solved through optimisation of the scanning parameters prior to the scan, based on the expected flow rates, for example, increasing the distance between the tags or decreasing the delay time between the application of the tag lines and acquisition of the image in order to capture higher velocity flows. In previous studies, MR tagging has been used to measure flows that are magnitudes stronger [33], using a single tag. Therefore, the use of a multiple tagged scan to identify regions of extreme flow due to erratic motility with pathological roots, followed by the application of a single tag to quantify the extent of the flows, could form a valuable clinical or research tool.

Learnings from the in vitro model can inform in vivo studies, if the biorelevance of the motility patterns explored using the DCM is understood. Recent cine-MRI studies have shown that antegrade contractile waves in the unstimulated human AC propagate at 0.98 cm·s^−1^ [22]. Since this is comparable to the CPPW applied to the DCM in this in vitro study, the tagging technique may be well-suited to velocimetry of the colonic contents during unstimulated contractile activity in the human AC. However, when stimulated, a 2.2-fold increase in the propagation velocity was observed in the AC, with antegrade and retrograde waves propagating at 2.2 ± 3.3 cm·s^−1^ and 2.2 ± 1.8 cm·s^−1^, respectively. This is a faster propagation speed than the CPPWs in this study, although similar to previous motility patterns simulated using the DCM [22]. Therefore, since different flow velocities can be expected from either faster or slower motility patterns inside the DCM, and whether the AC is stimulated or unstimulated in vivo, parameters, such as tag spacing and the delay between tag application and image acquisition, should be tailored accordingly to facilitate optimum tagging velocimetry.

### 3.3. Velocimetry of Fluid Motion In Vivo Using MR Tagging

**Figure 6:**
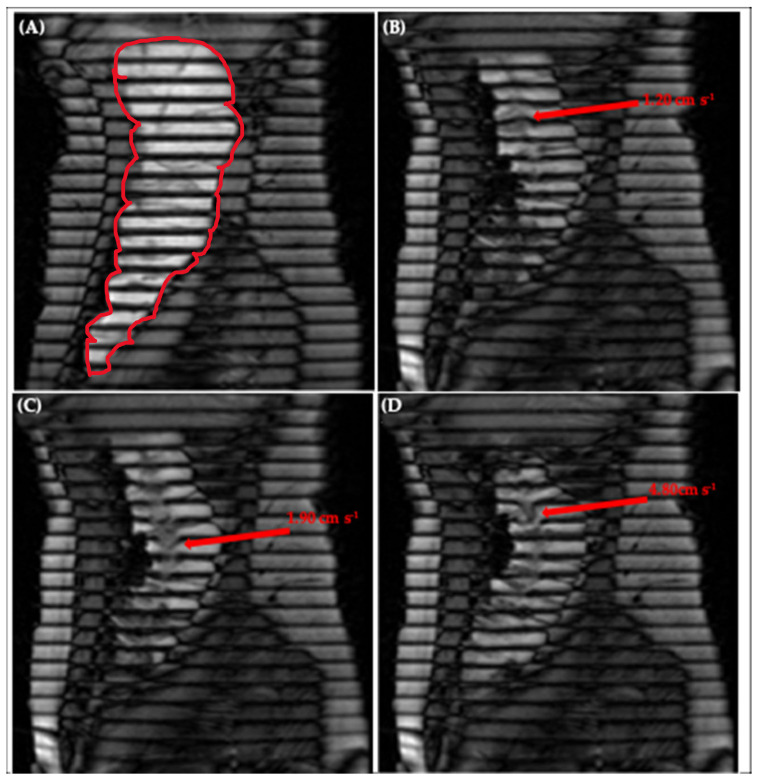
Tagging applied to the ascending colon, outlined in red in (**A**), showing (**A**) little motion, (**B**) antegrade flow of 1.20 ± 0.20 cm·s^−1^ close to the hepatic flexure, (**C**) retrograde flow of 1.90 ± 0.20 cm·s^−1^ close to the midpoint of the AC and (**D**) retrograde ‘jet’ of 4.80 ± 0.20 cm·s^−1^ close to the hepatic flexure. Images taken from supplementary video S2 of Pritchard et al. [20].

Figure 6A shows an image of the human ascending colon under baseline conditions with little to no motion, illustrated by the straight, parallel tags [20]. Figure 6B–D depicts a chronological selection of screenshots from Video S2, an in vivo tagged MRI sequence previously published by Pritchard et al. [20]. The opening sequence in video S2 begins with a contraction of the colonic walls close to the caecum, driving an antegrade wave of motion of the contents, with a residual velocity of 1.20 ± 0.20 cm·s^−1^, clearly shown further along the colonic axis in Figure 6B. Subsequently, the walls of the colon relax, dilating the lumen and driving the contents back towards the caecum in a wave of backflow first observable closer to the midpoint in the early frames of Video S2 and in Figure 6C and peaking with the following sharp jet of 4.80 ± 0.20 cm·s^−1^ shown in Figure 6D. The correlation between wall motion and flow events observed in this in vivo sequence is reproduced well in the DCM, as shown in Figure 2 and discussed in Section 3.1. This bolsters the DCM as a tool for enhanced biorelevant dissolution testing and demonstrates its suitability for the development of methods capable of achieving IVIVCs or performing IVIVE for colon-targeted formulations.

Thus, measurement of velocity of the tagged colonic contents under stimulated conditions in vivo was possible, with many tags remaining intact, permitting local velocity measurement at different spatiotemporal locations along the colonic axis. This facilitated the evaluation of regional and temporal similarities and differences. For example, both antegrade (1.20 ± 0.20 cm·s^−1^) and retrograde (4.80 ± 0.20 cm·s^−1^) flows were measured close to the caecum, whilst close to the midpoint of the human AC, retrograde flows of 1.90 ± 0.20 cm·s^−1^ were recorded. Similar antegrade and retrograde activity has been reported in vivo by [30]. In conjunction with visualisation of how the walls moved in real time, this methodology therefore has the potential to further inform an understanding of colonic pathophysiology. A clear application would be to establish quantitative limits that identify regions suffering from dysmotility based on the magnitude of the velocity of the contents compared to values observed in the healthy AC. A key advantage of the tagged MRI technique is the coverage acquired—the ability to both visualise and measure flows across the entire AC in one scan.

Complex multidirectional flows were observed under stimulated in vivo conditions, manifesting as smearing of the tag lines, and simultaneous antegrade and retrograde flow of media at different points along the same tag, as shown in Supplementary Materials Video S2 and Figure 6C,D. These phenomena were not observed in the DCM, where tagged media typically flowed in a laminar antegrade or retrograde fashion. The DCM motility was programmed to replicate CPPWs in the healthy human colon, as opposed to the extreme stimulated conditions observed in the in vivo study of [20]. This involved a cyclic repetition of one antegrade CPPW in which all haustra in a segment contracted or relaxed in a specific, predefined order and with a fixed degree and rate of occlusion. In vivo, motility is governed by the enteric nervous system which results in a more complex motion than the controlled wall motility of the DCM. However, the in vivo motility observed by Pritchard et al. [20] included both antegrade and retrograde waves, with haustra contracting and relaxing asynchronously and with different degrees of occlusion, which due to the momentum of the contents, caused more complex multidirectional flows. Similar activity has been observed in vivo by Wilkinson-Smith et al. [16], where contractions in the AC wall were not continuous, but sporadic. Multidirectional motility patterns could be replicated in future work with the DCM to more precisely mimic specific in vivo patterns for direct comparison. Furthermore, there are many other factors associated with administration of the stimulus that may affect the motility in vivo. For example, the rate of delivery of macrogol to the AC, the mixing of the macrogol with the contents of the colon upon arrival and the absorption rate of any fluid from the macrogol [16]. These factors, in addition to characteristics of the patient such as age [40], influence the volume of the contents of the AC, which is likely to trigger distension of the walls in vivo, hence driving the erratic motility. In vitro, the DCM was only partially filled (150–200 mL, 52–69%), compared to the AC which was full of contents after the macrogol stimulus.

The complex flows caused blurring of the tag lines which can break in places, rendering the tagging method of flow quantification invalid. Ultimately, tagged MRI failed to accurately measure velocities at all tagged locations in extreme conditions in vivo, but was still able to measure velocities at individual tagged locations where the tags remained intact. However, it must be appreciated that these extreme conditions are among the least abundant motility types observed naturally in vivo, simply meaning that this technique forfeits its applicability to measure intracolonic flows under extreme conditions generated by stimulation or that may occur naturally in patients presenting with certain disease states, such as diarrhoea [30]. Nevertheless, this field is currently in its infancy and therefore revealing the upper limits of this technique is additional added value resulting from this work.

Due to the apparent randomness of the starting point for a propagating contraction in vivo, visible in the in vivo sequence (Supplementary Materials Video S2) and high-resolution manometry studies [30], it is important that preliminary scans apply multiple tags across the colonic axis, as in this study. However, as previously mentioned, the application of a single tag is useful when high flow velocities are expected. Therefore, single tag velocimetry analysis of areas already identified as having extreme motility from a prior multi-tag scan, could add value in a clinical or research setting by quantifying the degree of extreme flow in patients presenting with a motility-related pathology.

### 3.4. PC Cine-MRI Velocimetry of the DCM Lumen Contents

**Figure 7:**
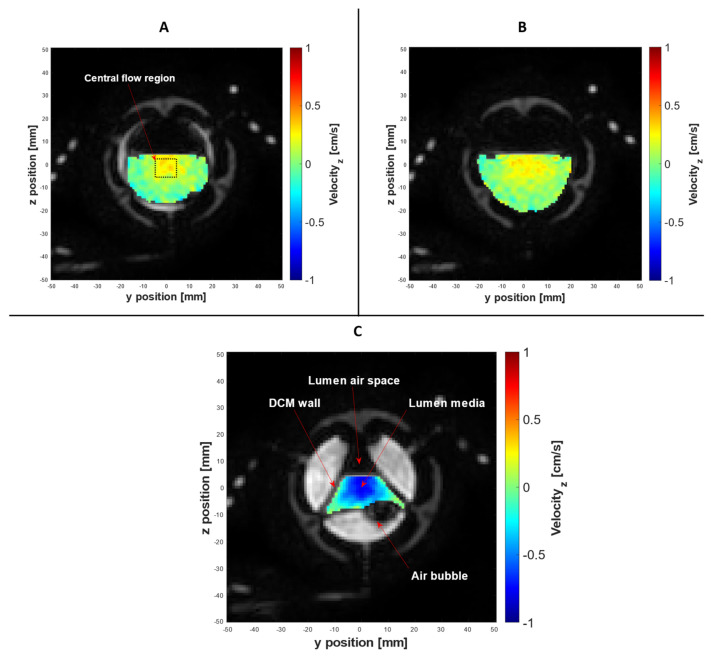
PC cine-MRI of the DCM, with velocity encoded in the *x*-axis. Simultaneous acquisition of morphological data and spatially registered flow at the cross section of tag 11, placed at the midpoint of segment 6. Parts **A**–**C** were produced by superimposing the two data sets at different stages of motility: neutral, during upstream motility (7**A**), relaxation (7**B**) and contraction (7**C**).

Figure 7 presents a selection of morphological images with the associated spatially registered through-plane velocity maps superimposed over the lumen media, acquired using PC cine-MRI at the midpoint of segment 7, the cross section at Tag 11, and at different temporal locations. As highlighted in a recent review of in vitro models of the GI tract, the DCM is currently the only in vitro model to replicate peristaltic motility in a lumen with the segmented architecture of the human colon [23]. Therefore, understanding the streamwise velocity profile through a segment of the DCM at different stages of biorelevant motility adds insights to how intestinal wall motion influences the hydrodynamics that drive dissolution and mixing. The box drawn at the centre of Figure 7A outlines the area represented by the central flow region of the lumen. The idea of combining tagging and PC cine-MRI, for the acquisition of in-plane and through-plane motion information, has been explored previously by Perman [41] and Kuiljer [42].

To account for the background signal, initial velocity measurements were taken using PC cine-MRI prior to any induced motility (neutral wall position) when it was known the luminal contents were at rest. The mean velocity over the cross-sectional lumen flow area was close to zero at 4.32 × 10^−4^ cm·s^−1^ with a standard deviation of 6.40 × 10^−3^ cm·s^−1^. This standard deviation value was taken as the measurement error for a single voxel and hence the error for PC cine-MRI peak velocity measurements, and is three orders of magnitude smaller than the expected maximum signal (± 3 cm·s^−1^).

Overall, the morphological information and associated velocity maps acquired using PC cine-MRI accurately explained the flow phenomena in the tagged contents described in Figure 4. Figure 7A shows the cross section at tag 11 at *t* = 8.0 s. The DCM walls were at the neutral position and a low antegrade flow was focused around the centre of the lumen toward the free surface, in keeping with the positive peak in cyclic antegrade–retrograde flow recorded by the tagged dataset in Figure 4 at *t* = 8.0 s. Due to the neutral position of the DCM walls, it can be confirmed that this flow was caused by the upstream wall displacement shown in Figure 5. There are clearly stagnant regions close to the walls. This suggests that any dosage form agglomerates that lie in the stagnant corners may not experience high peaks in shear forces arising from the flow; however, the proximity to the walls is likely to result in elevated normal forces exerted on the dosage form during contraction, which may accelerate break up and drug release. This kind of squeezing force is absent in typical pharmacopeial dissolution apparatuses, but is relevant for colon-targeted dosage forms due to the architecture of the colon and its wall movements.

Figure 7B shows the cross section of tag 11 at *t* = 28 s, depicting the walls in a fully relaxed state, maximising the cross-sectional area of the lumen. This is in line with the beginning of an antegrade flow episode observed in the tagged dataset (Figure 4A). Flow was in the positive *x*-direction, driven by the immediately upstream DCM contraction, in keeping with the antegrade flow event recorded in the tagged contents. Again, the flow was concentrated in the centre of the lumen close to the free surface. During the relaxation of the walls immediately before the image in Figure 7B, the “pouring mode” described by Alexiadis et al. [43] may occur where significant flows in the *z*-direction take place; this is not shown, as velocity was encoded only in the *x*-direction. The tagged dataset in Figure 4 shows a strong peak in negative velocity at *t* = 35.4 s, aligning with the cross section captured in Figure 7C which shows the walls fully contracted at *t* = 36.0 s, occluding the lumen and forcing the contents backwards. An established velocity profile is evident, with the highest retrograde velocities in the centre of the lumen and steepest velocity gradients towards the walls.

The combination of antegrade flow and the “pouring mode” during relaxation of the walls, followed by occlusion of the lumen and strong backflow may subject dosage form agglomerates and the released API to stresses and mixing phenomena unique to the intestinal lumen. This is likely to result in tablet–liquid mass transfer coefficients that are highly dependent on the tablet location with respect to contractions of the mimic colonic walls and also on the frequency of contractions. Since the motility and architecture of the DCM are based on human anatomical data, this is probably a much closer representation of the in vivo situation than in typical pharmacopeial systems. For example, the USP2 and USP mini vessels, which typically operate under continuously mixed conditions using a paddle, form two flow loops above and below the paddle, with the tablet sitting in a relatively stagnant zone beneath the paddle [7,27,44]. The parabolic flow profile during pulsatile flow in the flow through cell (FTC), another internationally recognised pharmacopeial dissolution apparatus used to assess modified-and extended-release dosage forms [45], bares some semblance to flow in the partially filled DCM. However, the two are not comparable due to the many dissimilarities including (but not limited to) phenomena common in partially filled pipes, such as the velocity drop phenomenon [46], vessel architecture, multidirectional flows due to the nature of peristaltic waves, and a dissimilar (higher) pulse rate in the FTC compared to a physiologically-relevant rate of peristaltic contractions in the DCM [22,30].

**Figure 8:**
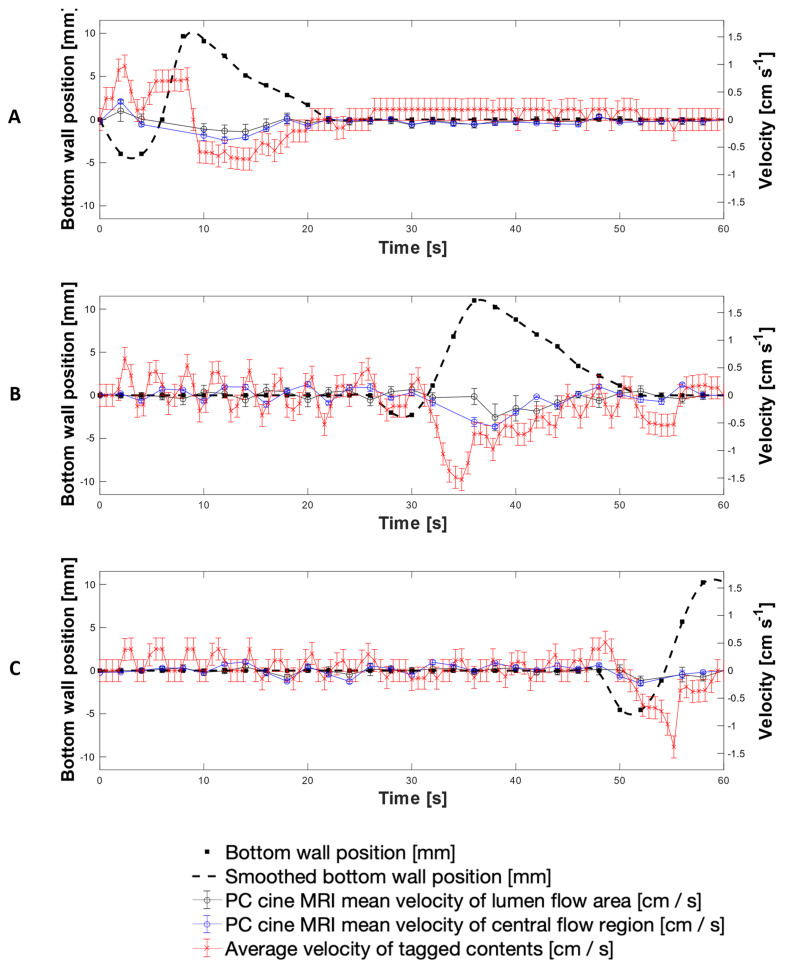
Average and mean velocities measured using tagged and PC cine-MRI at the location of tags 3 (**A**), 12 (**B**) and 19 (**C**), alongside wall displacement at the corresponding location (dashed line).

Figure 8 presents a direct comparison of velocity data collected using both the PC cine-MRI technique and the tagging technique using 200 mL LOVIS fluid subjected to the 0.4 cm·s^−1^ CPPW at the location of tags 3, 11 and 19. Additionally, wall motility was quantified by displacement from its neutral datum as shown by the black dashed line, measured by tracking the wall membrane in the magnitude component of PC cine-MRI images. This permitted tracking of the fluid velocity with respect to the wall motion and thus the consequences of the wall motility on the motion of the contents, to be evaluated directly. Comparison of the adjacent plots in Figure 8 facilitates visualisation of how the contents moved as the contractile wave propagated along the *x*-axis of the DCM tube.

Since the measurements were taken on different scans, the wall waves and therefore fluid flows were marginally out of phase, but overall, the average velocity-time profiles measured using the tagged methodology and the mean PC cine-MRI measurements were similar. Velocities measured using tagging were consistently higher in magnitude than the mean PC cine-MRI measurements; however, PC cine-MRI mean velocity measurements using the central flow region closely aligned with the tagged methodology. This shows that the average velocity of tagged contents more closely represented flow of the central plug of media enclosed by the region outlined in Figure 7A, rather than representing flow across the entire cross section at the tagged location. This, alongside the consistently higher velocity measurements, was likely to be due to the difference in the 2-dimensional plane in which measurements were taken and the discrete values that the tagging velocities were restricted within. PC cine-MRI measures flow through a cross section perpendicular to the flow, and therefore consideration of the entire luminal cross section involves a greater voxel coverage of stagnant regions towards the walls of the lumen where lower flows are abundant. Voxels containing these low flows were less significantly represented in the displacement of the centroid of a tag, compared to in PC MRI mean velocity estimation. This is because tag displacement is analysed in the 2D longitudinal axis, where voxels containing more extreme non-transversely uniform flows have an equal weight to a voxel containing a region that is stagnant through a larger proportion of the transverse plane. Therefore, the nature of the tagging technique affords it a higher intrinsic sensitivity to flows along the *x*-axis of the DCM. A deeper analysis of tagging versus PC cine-MRI measurements is presented in Appendix A, whilst Appendix B explains the absence of PC cine-MRI data points.

The highest antegrade average flows were observed at the location of Tag 3 (shown in Figure 8A), close to the start of the CPPW, recorded as 0.90 ± 0.20 cm s^−1^ at *t* = 1.8 s using tagging, compared to 0.16 ± 0.19 cm·s^−1^ and 0.33 ± 0.04 cm·s^−1^ using PC cine-MRI measurements of the entire lumen and the central flow region, respectively at *t* = 2 s. After the local wall segment returned to the neutral position, all velocities remained approximately stable between the limits of −0.20 cm·s^−1^ and 0.20 cm·s^−1^. At the location of tags 11 and 19, fluctuation in the velocity prior to local wall motility consistently measured between approximately −0.50 to 0.50 ± 0.08 cm·s^−1^ and −0.20 to 0.40 ± 0.08 cm·s^−1^, respectively. Average retrograde velocities consistently peaked immediately before or during local wall motility, with the fastest average retrograde velocity of −1.53 ± 0.20 cm·s^−1^ at the location of tag 11 (shown in Figure 8B) at *t* = 34.8 s, compared to −0.40 ± 0.24 cm·s^−1^ and −0.57 ± 0.02 cm·s^−1^ at *t* = 36.0 s for PC cine-MRI analysis of the whole lumen flow area and central flow region, respectively. This finding is in line with CFD modelling of the colon by Sinnot et al. [47], which found that higher speed retrograde flow is visible local to the contracting region and extends further upstream than the contraction. Overall, velocities in the DCM during the 0.4 cm·s^−1^ CPPW were much lower than in the pharmacopeial USPII and mini vessel, where they were 50–100 rpm and 50–200 rpm, respectively, and tangential velocities have been reported to reach 15 cm·s^−1^ [7,44]. On the other hand, the maximum velocities observed in the FTC are far lower than in the DCM, not breaching 0.4 cm·s^−1^ even at the highest flow rate typically used in pharmacopeial dissolution testing (16 mL min^−1^) [45].

**Figure 9:**
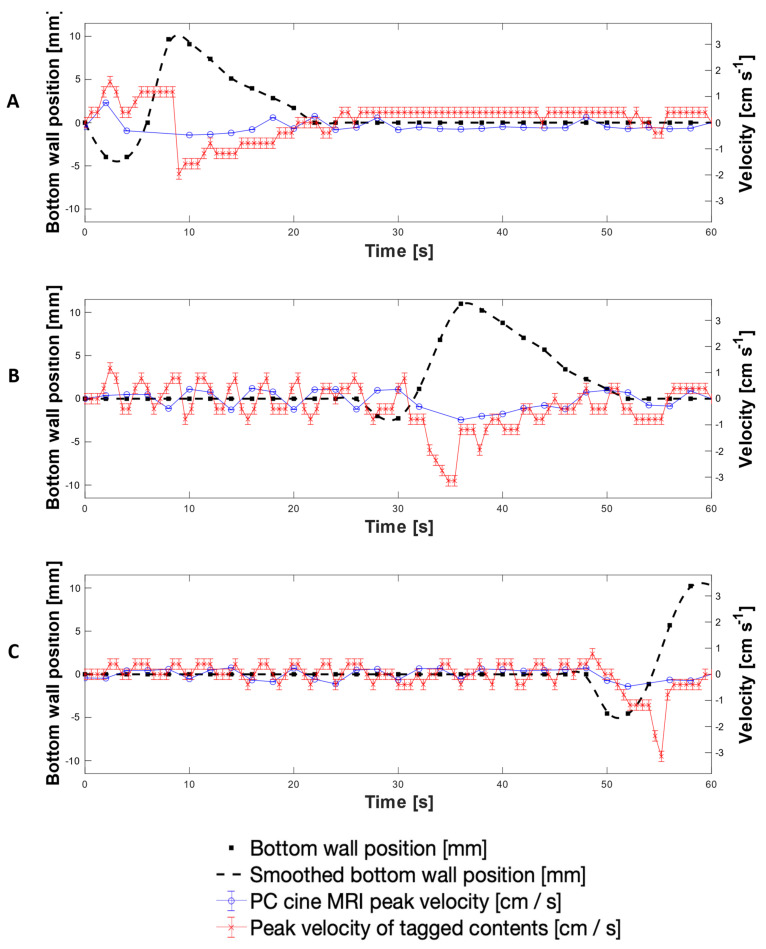
Peak velocities recorded using tagged and PC cine-MRI at the location of tags 3 (**A**), 12 (**B**) and 19 (**C**), alongside wall displacement at the corresponding location (dashed line).

Figure 9 shows the peak velocities recorded using each MRI technique under the same experimental conditions as Figure 7. Typically, the peak velocities followed the same trend as the average velocity measurements and there was good agreement between tagging and central flow region PC cine-MRI peak velocity measurements. However, tagging consistently registered higher velocity measurements where highly localised regions of fast-moving media (typically around *z* = 0) caused sharp displacement of a tag. Voxel intensity is proportional to the volume of media displaced which varies along the *z*-axis, as shown by the velocity profiles in Figure 7. The highest antegrade velocity recorded was 1.60 ± 0.20 cm·s^−1^ in tagged data and 0.76 ± 0.20 cm·s^−1^ in PC cine-MRI; both faster than the speed of the propagating wave, 0.4 cm·s^−1^. Previously in positron emission particle tracking (PEPT) studies, Stamatopoulos et al. [22] recorded maximum antegrade velocities (2.20 cm·s^−1^) using a faster propagating wave (2 cm·s^−1^). Previous in vivo analyses of colonic motility using a magnetic pill revealed a broad spectrum of velocities [48,49]. However, interpreting motion of the colonic contents from the observed motion of a tracer particle must be done carefully since the particle relaxation time can inhibit its ability to follow the fluid. This makes direct comparisons between datasets, either in vivo or in vitro, difficult [22]. Noninvasive methods such as MRI have a significant advantage in this regard.

Retrograde velocities inside the DCM have previously been reported to reach 4 cm·s^−1^ using 0.25% (w/v) NaCMC in previous PEPT studies [22]. The highest retrograde velocity observed in the DCM in this study was similar; −3.10 ± 0.20 cm·s^−1^ at *t* = 55.8 s during local contraction at tag 19 toward the later stage of the CPPW. This was likely to be influenced by gravitational forces as media rises up the hepatic flexure but does not cross it, causing it to fall back into the lumen with a strong backflow. This measurement is in line with the in vivo observation in Figure 6D, wherein the highest measured retrograde velocity (4.80 cm·s^−1^) occurred close to the hepatic flexure during relaxation of the colonic walls after a propagating contractile wave. Similarly, this could be due to a combination of the influences from relaxation of the walls and gravitational forces. The highest average retrograde velocity, −1.50 ± 0.20 cm·s^−1^, was observed at the position of tag 11 just past the midpoint of the DCM. Comparatively strong retrograde velocities in segment 2 and low antegrade velocities help to explain why Stamatopoulos et al. [21] observed theophylline extended-release dosage forms remaining closer to the *caecum* throughout dissolution experiments, with the highest drug release rates measured close to the *caecum* compared with downstream sampling points. These findings show ineffective aboral transport at early stages of the CPPW close to where a solid oral dosage form would enter the colon through the ileocaecal valve, consistent with previous findings which demonstrated poor overall propulsion of contents using low viscosity media [27].

The findings in this work strengthen conclusions made by previous studies [16,20], that combining this technique with existing motility and volume measurements could improve classification between healthy subjects, and subjects whose colonic motility is absent or ineffective at mixing and transporting the contents. This has the potential to facilitate stratification of the functional type from irritable bowel syndrome (IBS) type disorders and contribute to the development of an understanding of local intracolonic environmental conditions, which are essential to optimise the delivery of therapeutics for patient-centred formulation design. However further investigation in such patient groups is needed to explore this possibility.

## 4. Conclusions

This work demonstrates that a noninvasive MR tagging method developed to assess human colonic motility and distinguish between colonic disease states, can differentiate between motility patterns and media viscosities in a biorelevant in vitro model of the human ascending colon. This adds value to the MR tagging technique in its ability to understand colonic pathophysiology, the effectiveness of treatments and the conditions that a colon-targeted dosage form may experience in vivo.

The same tagged dataset was used to directly measure the velocity of the luminal contents inside the in vitro model and a tagged sequence of the ascending colon in vivo, with simultaneous knowledge of how the walls move. It was demonstrated that the dynamic colon model (DCM) can induce flow that replicates the flow patterns observed in vivo, of a similar velocity magnitude. To reproduce the hydrodynamics of the human ascending colon is an essential capability for a biorelevant mechanical dissolution apparatus to be suitable for the development of methods facilitating IVIVE or IVIVC.

PC cine-MRI revealed the streamwise flow profiles observed inside the DCM lumen, which represents the segmented architecture of the ascending colon, at different stages of a biorelevant peristaltic wave. Flow profiles resembled flows in a partially filled pipe, but differed greatly to those observed in compendial dissolution apparatuses, such as USP2 and the flow through cell (USP4). Nevertheless, this visualisation elucidates the environment that a dosage form may be subjected to inside the DCM during dissolution studies which is vital in understanding how the motion of the mimic colon walls drive the hydrodynamic conditions that govern the erosion of a dosage form and the release and dissolution of the API. Additional insights into colonic flow gained through the model could be useful to inform in silico modelling of colonic drug release and dissolution.

## Figures and Tables

**Table 1:** Imaging parameters.

Parameter	DCM PC cine-MRI	DCM Tagging	Human Colon Tagging [20]
Scan duration [s]	60	60	20
TR [ms]	9.21	2.43	2.30
TE [ms]	7.60	1.22	1.15
FA [°]	10	45	45
FOV [mm^2^]	177 × 200	259 × 330	222–264 × 330
Recon resolution [mm^2^]	1.136 × 1.136	0.982 × 0.982	0.982 × 0.982
Slice thickness [mm]	8	15	15
SENSE	2.0	1.5	1.5
No. dynamics	30	100	33
Temporal Resolution [s]	2	0.6	0.6
No. averages	1	1	1
Orientation	Transverse	Sagittal	Sagittal

**Table 2:** Degree of mixing (CoV) scores reported from tagging experiments and Tukey’s post-hoc assessment for significant differences (*n* = 4). All interaction effects were deemed insignificant (*p* > 0.05) so were not tabulated.

Media		Volume	CoV (%)
**Slower CPPW (0.4 cm·s^−1^)**			
LOVIS		150 mL	32.16
		200 mL	30.23
HIVIS		150 mL	26.52
		200 mL	25.68
**Faster CPPW (0.7 cm·s^−1^)**			
LOVIS		150 mL	37.29
		200 mL	37.42
HIVIS		150 mL	32.35
		200 mL	31.98
**Post-hoc multiple comparisons of CoV using Tukey’s HSD test**			
**Variable**	**Level difference**	**Mean difference**	***p*-value**
Motility pattern	Slower–Faster CPPW	6.113	*p* < 0.05
Media viscosity	Media LOVIS-HIVIS	5.142	*p* < 0.05
Media volume	150–200 mL	−0.752	*p* < 0.05

## Data Availability

The data that support the findings of this study are available on request from the corresponding author.

## Supplementary Materials

The following are available online at https://www.mdpi.com/article/10.3390/pharmaceutics13101545/s1, Video S1: In vitro tagging scan. Video S2: In vivo tagging scan.

## Appendix A

### Scrutininsation of Tagging versus Phase Contrast Cine-MRI Velocity Measurements

MR tagging has been used to measure velocity in fluid flows, primarily in blood vessels [17] and more recently in engineering systems [18,19]. In both applications however, flows have typically been much faster than those previously measured inside the DCM (which were of the order of 2–4 cm·s^−1^ [22]) and mostly occurred within far smaller geometries, such as veins or nozzles, and usually with the application of only a single tag. Phase contrast (PC) cine-MRI, described in detail in [31], is also used to measure blood flow [32]. However, PC cine-MRI has also been applied to measure lower velocity cerebrospinal fluid (CSF) flows which are commonly of the order 0.5–5 cm·s^−1^ [50,51,52,53,54] and therefore much more comparable to flows inside the DCM. Moser et al. [18] compared both techniques to study flow inside a pipe with a step stenosis. It was found that PC cine-MRI gave more accurate results than tagging techniques for flows of <100 cm·s^−1^. Contrarily, d’Avila found that tagging techniques made accurate measurements for flows as low as 0.1 cm·s^−1^ inside a concentric cylinder system [19].

The precision of the measurements must be scrutinised by delving into the characteristics of the tagged technique, since measurements inside the DCM were consistently higher than those found using PC cine-MRI. Tagged methods have been reported to measure velocities accurately in phantoms [18,19,33,55], including low flows [19], pulsatile flow [55], and in vivo [55]. Contrarily, when compared with PC cine-MRI, Moser et al. [18] found that only 50 and 79% of tagged velocity measurements agreed, to within 10% of CFD velocity measurements for Reynolds numbers of 100 and 258, respectively, compared to 90 and 94% for PC cine-MRI. Flows studied were between 0 and 10 cm·s^−1^, similar to flows inside of the DCM. To minimise errors in future tagged experiments, small evolution times can be chosen since the estimated velocity of a tag is given by u_x_ = ∆x/∆t; however, this causes the error in measuring displacement to become more significant. Additionally, a decrease in the evolution time at a constant voxel size has been shown to improve accuracy of tagged velocity measurements from 0.30 m·s^−1^ to 0.13 m·s^−1^ [33]. PC cine-MRI has been reported to be more accurate than tagged TOF for steady, laminar Poiseuille flow [56]. Moreover, Moser et al. [18] similarly used both techniques to measure velocities of a similar magnitude (0–10 cm·s^−1^) in a pipe with a step stenosis. For slower velocities, PC cine-MRI gave more accurate results compared to numerical CFD solutions of velocity. Therefore, it may be hypothesised that the tagged TOF methodology has overshot the velocity measurements inside the DCM and therefore, potentially inside the human AC too. Further, problems arose from the blurring of tag lines. In addition to complex flows, blurring of tags lines can happen at elevated flows due to an influx of spins from outside the radiofrequency (RF) coil entering the FOV during the tag evolution time [18].

It is unsurprising that PC cine-MRI may yield more accurate velocities than tagging, considering the tagged method utilises a point-based data reduction method used to extract velocity, which is essentially a hand-measured velocity extraction technique. In contrast, PC cine-MRI does not require such an intermediate step; it directly provides a velocity map of the lumen wherein each voxel value represents the weighted average velocity measured in that voxel.

However, the same study by Moser et al. [18] found that the largest discrepancy between PC cine-MRI and CFD measured velocities occurred just downstream of the sudden contraction of the stenosis. Here, PC cine-MRI measured velocities were lower than the numerical predictions towards the centre of the pipe. This suggests that the similar contractile behaviour inside the DCM may have led to underestimations on the part of PC cine-MRI, rather than overestimations from tagging. Similarly, Ahmadi and Mastikhin [33] found that phase encoding (MS SPRITE) velocity measurements underestimated velocity, whilst tagged velocity measurements were more accurate. Underestimated velocity measurements are consistently reported in the literature when the TE has been high. For example, O’Brien et al. [34] found significant errors in velocity measurements in in vitro stenotic flow, with large underestimates of velocity that reduced with a shorter TE: average error: –166%/–67%/–25%/–13%/–8.8% for TEs of 4.8/4.0/3.3/2.2/2.0 ms. This study used a comparatively high TE of 7.6 ms which could explain the large discrepancies between the tagged and PC velocities during contractions of the DCM walls, with PC measurements consistently lower than those of tagging. However, it is appreciated that the studies are not entirely comparable since O’Brien et al. [34] studied flows of up to 900 cm s^−1^.

Furthermore, the need to develop methods of extracting velocity fields from spin-tagging images, due to the drawbacks of PC cine-MRI, has been emphasised in the literature [56]. PC cine-MRI assumes that velocity does not vary significantly across each voxel. In the case of complex 3D flows or regions of high shear, this assumption is violated. Due to the level of complex flows observed in the stimulated AC in vivo, PC cine-MRI is unlikely to make reliable measurements. Furthermore, although PC cine-MRI can measure slow and fast flows, its dynamic range is limited by the signal-to-noise ratio, because the flow-encoding gradient must be limited to avoid phase wraparound. PC cine-MRI is also susceptible to error from phase accumulation caused by fluid acceleration, velocity averaging over the slice thickness, spin flow-through and magnetic susceptibility differences between the media and the walls [18]. Considering in vivo applications, PC cine-MRI is sensitive to bulk motion, which can lead to difficulties associated with respiratory motion [20]. MR tagging is also sensitive to bulk motion but is advantageous as tag lines can easily be tracked outside of the colon to help differentiate bulk from local motion. Secondly, tagging also provides a greater spatial coverage of the colon compared to PC cine-MRI, although there are limitations on the direction of flow that can be quantified (perpendicular to the tag line placement only). Lastly, PC cine-MRI loses sensitivity if the T2 of the sample is short, as the signal will decay during the phase encoding period, whilst tagging loses sensitivity if the T1 of the sample is too short, as the tags will recover (and hence disappear) in the delay period. These properties are likely to favour the use of tagging over PC cine-MRI based on the MR properties of normal human colonic contents.

A critical advantage of the tagging technique in this study was the temporal resolution of 0.6 s compared to the poorer resolution of 2 s for PC cine-MRI. This meant additional measurements from tagging, between the PC cine-MRI measurements, could be detected. This revealed peak velocities during local wall motility, where flow was rapidly evolving and fluctuating. Improved temporal resolution is achievable with PC cine-MRI, at the cost of decreased sensitivity to flows inside the DCM and lower spatial resolution, which is important to precisely track wall motion and synchronise this with the incurred flows. Typically, the temporal resolution for PC blood velocity measurements is ≤ 50 ms [57]. PC cine-MRI can often be limited by temporal resolution when studying slow flows because the slower the velocity encoding, the stronger the magnitude of bipolar gradients that must be applied for a longer period of time, thus increasing TR. On the contrary, TR cannot be indefinitely increased, as a relatively short TR is required to achieve maximal temporal resolution [58].

## Appendix B

### Exclusion of Phase Contrast Cine-MRI Datapoints

The image taken immediately prior to maximum contraction was discarded in all PC cine-MRI scans, due to intravoxel dephasing, resulting in signal loss and therefore velocities were unable to be measured with any degree of certainty. This could be due to higher order motion, i.e., acceleration, [59] or turbulence [60,61]. Although the flow regime inside the DCM is not considered to be turbulent, complex hydrodynamics are expected to occur due to the pulsatile, unsteady nature of peristalsis, such as the ‘pouring mode’ described by Alexiadis et al. [43]. Ståhlberg et al. demonstrated that intravoxel dephasing can be combatted by shortening the TE [59]. This phenomenon was absent in the tagged methodology which captured viable data at each time point.
